# Mental Peculiarities and Mental Disorders of Childhood

**Published:** 1860-09

**Authors:** Charles West


### MENTAL PECULIARITIES AND MENTAL DISORDERS OF CHILDHOOD.

BY DR. CHARLES WEST.

No one can have watched the sick bed of the child without being struck with the almost unvarying patience with which its illness is borne, and the extremity of peril from which, apparently in consequence of that patience, a complete recovery takes place. Much, indeed, is no doubt owing to the activity of the reparative powers in early life, but much also to the unruffled quiet of the mind. No sorrow for the past, no gloomy foreboding of the future, no remorse, disappointment, nor anxiety depresses the spirits and enfeebles the vital powers. The prospect of death, even when its approach is realized, and this is not so rare as some may imagine, brings in general but small alarm; it may be from the vagueness of the child's ideas; it may be, as the poet says, that in his short life's history "the heaven that lies about us in our infancy" has been so much within him, that he recognizes again more clearly than we can do—

> "The glories he hath known,
> And that imperial palace whence he came."

I refer to this, gentlemen, because the truth is one which has its practical side; because to keep the sick child happy, to remove from it all avoidable causes of alarm, of suffering, of discomfort, to modify our treatment so as to escape a possible struggle with his waywardness; and even, if death seems likely to occur, to look at it from a child's point of view, not from that which our larger understanding of good and evil suggests to our own minds, are duties of the gravest kind, which weigh on the physician, on the parent, on the nurse; and which it behooves to remember none the less, because they are not dwelt on in the lecture room, or in the medical treatise.

But not only does the child live in the present far more exclusively than is possible for the adult, but there are, besides, other important points of mental difference between the two which have a serious influence on the manifestations of disease, and also on our treatment of it. The mind of the child is not merely feebler in all respects than that of the adult, but in proportion to the feebleness of his reasoning powers, there is an exaggerated activity of his perceptive faculties, a vividness of his imagination. The child lives at first in the external world, as if it were a part of himself, or he a part of it, and the glad-heartedness which it rejoices us to see is as much a consequence of the vividness with which he realizes the things around him, as of the absence of care to which it is often attributed. This peculiarity shows itself in the dreams of childhood, which exceed in the distinctness of their images those which come in later life, and shows itself, too, in the frequency with which, even when awake, the active organs perceive unreal sounds, or conjure up at night ocular spectra; and these not merely colors but distinct shapes, which pass in long procession before the eyes. This power fades away with advancing life, until, except under some condition of disease, the occasional appearance of luminous objects in the dark, remains the only relic of this gift of seeing visions, with which, in some slight degree, at least, most of us were endowed in our early years. The child who dreads to be alone, and asserts that he hears sounds or perceives objects, is not expressing merely a vague apprehension of some unknown danger, but often tells a literal truth. The sounds have been heard. In the stillness of its nursery the little one has listened to what seemed a voice calling it; or, in the dark, phantasms have risen before its eyes, and the agony of terror with which it calls for a light, or begs for mother's presence, betrays an impression far too real to be explained away, or to be suitably met by hard words or unkind treatment.

Impressions such as these are common in childhood even during health. Disorder, direct or indirect, of the cerebral functions, more commonly the latter, greatly exaggerates them. The minor degrees of somnambulism, such as getting out of bed while asleep, are by no means uncommon in childhood, and even more frequent are those attacks of night-terrors, in which, after a short doze, the child awakes in a state of intense alarm, with the distinct vision before it of some frightful object, which does not disasappear for some minutes, and which returns sometimes the same night, sometimes the succeeding one, with just the same appearance as before.

It follows, then, that the circumstances which surround a child whether in sickness or in health, are of far more importance than are those about the adult; and that their influence for good or for harm, is far more powerful, and is never to be lost sight of in the treatment of the diseases of early life.

But while the child lives thus in the present, and while this present is but the reflection of the world around, its impressions uncontrolled by experience, ungoverned by reason, the moral qualities are not in the same undeveloped state as the intellectual powers. The child loves intensely, or dislikes strongly; craves most earnestly for sympathy, clings most tenaciously to the stronger, better, higher, around it, or to what it fancies so; or shrinks in often causeless, but uncon querable dread, from things or persons that have made on it an unpleasant impression. Reason as yet does not govern its caprices, nor the more intelligent selfishness of later years hinder their manifestation. The waywardness of the most willful child is determined by some cause near at hand; and he who loves children and can read their thoughts, will not, in general, be long in discovering their motives and seeing through their conduct.

One word more I have to say with reference to that intense craving for sympathy so characterestic of the child. It is this which often underlies the disposition to exaggerate its ailments, or even to feign such as do not exist, and in which attempts at deception it often persists with almost incredible resolution. Over and over again I have met with instances, both in private and hospital practice, where the motives for such deception were neither the increase of comfort nor the gratification of mere indolence, but the monopolizing the love and sympathy which, during some by-gone illness, had been extended to it, and which it could not bear to share again with its brothers and sisters. This feeling, too, sometimes becomes quite uncontrollable, and then the child needs as much care and as judicious management, both bodily and mental, to bring it back to health, as would be called for in the case of some adult hypochondriac or monomaniac.

These mental peculiarities of early life may seem at first to have little to do with the cure of disease; but in reality you will find that this is not so; but that in proportion as you make them your study, and as you become able, in consequence, to sympathise more completely with your patient, will your diagnosis, in many instances, be more accurate, and your treatment more successful.

This brief sketch must suffice for the first part of my subject, and will, I trust, have prepared you for the better examination of the second, namely, the influence of disease upon the mind in early life. This shows itself either in weakening of the perceptive powers or in altering and perverting the moral faculties. Of these, sometimes the one, sometimes the other, is the more obvious, though it is very rarely that either exists absolutely alone.

As in diseases of the body, so in the affections of the mind, in early life, the power of repair furnishes us with a constant ground for hopefulness, which we should be less warranted in indulging in the case of an adult. The dullness, the apathy, the cerebral disturbance, which accompany the diseases of early childhood, have therefore by no means so grave an import as we should be compelled to attach to them, if present to the same extent at a more advanced age. The whole of the child's intellectual energy is expended on his commerce with the world around him; his relations to it are disturbed; night terrors, bad dreams, distressful phantasms, betoken this; or the ear is pained by sounds, and the eye by light,—not because the organs of sight or of hearing are specially disordered, or the brain is specially affected, but because, with the limited mental endowments of the child, such are the only ways in which their sympathetic disorder can manifest itself.

Or the disease has passed away; the active, intelligent, observant child is left dull, takes no interest in what goes on around him, forgets his prattle, seems scarcely to know the simplest word, though before he spoke fluently. This, again, need not cause too much apprehension. The child's memory is feeble; during the protracted illness, the customary impressions were no longer made upon his senses in the sick room, or they passed unnoticed in the unconsciousness of fever; so that, when recovery takes place, the lesson has to be learnt again, and learnt with faculties weakened by the by-gone ailment. For this you must be prepared, and prepared also for the very gradual process, extending sometimes over months or years, by which the ground lost is made good again: the time occupied by it being in general all the longer, in proportion as the child was younger when the original attack of illness came on. The infant of eight months old will show for months no ray of dawning intelligence; the little one just beginning to speak, will remain silent for months; while the child of four or five years will generally in a few weeks regain his forgotten lore. Simple as all this seems to be, and really is, it yet is not always borne sufficiently in mind; and these hints may enable you to save your patient's friends from much needless anxiety.

There is a caution, however, not to be lost sight of in these cases; namely, that protracted illness, even when unaccompanied by evidence of serious disorder of the brain, is sometimes succeeded by permanent impairment of the sense of hearing, and that the child's dullness may be the result of the loss of the power of receiving impressions, through one most important medium, from the external world.

A little girl, two years and nine months old, was attacked at the age of one year and nine months, by what was said to have been inflammation of the lungs, though it is uncertain whether or no a convulsion occurred at the outset. She got well without any other sign of cerebral disturbance having manifested itself. Before the illness she was beginning to talk, used to call her father, and to say many little words, but since then she had not spoken at all. The question raised was, whether the silence and some strange ways, different from the child's previous manner, were indicative of disease of the brain and of incipient idiotcy, or were merely the results of her loss of hearing. I took the child into the hospital for a time to watch her; her intelligent countenance and strangely earnest manner showed almost at once that her intellect was acute enough. A very few days' observation confirmed this impression; the child was dumb because she had become deaf, and her speech had ceased so soon, because the progress she had made at the time when illness first overtook her was so small.

If the same accident occurs somewhat later in life, when the child has learned to speak pretty well, its nature is more likely to be misunderstood, for the child does not all at once forget to talk, but speaks imperfectly, with gradually lessening distinctness; and forgetting first some words, then others, her condition more nearly resembles one of imbecility; and unless special care be taken to test the powers of hearing, error is very likely to be committed. I confess I made this mistake myself in the case of a little girl four years and seven months old, whom I first saw a year after an attack of a somewhat ill-defined febrile character, accompanied by a comatose condition, which lasted for several days, and left her in a state of great weakness. Though she regained strength, it yet was a long time before she tried to speak again; and when she did, her articulation was very indistinct. In the course of time she seemed as strong as ever, and her intelligence appeared not deficient, but her utterance grew more and more indistinct; she became flushed, angry and excited, and made inarticulate noises when she could not obtain what she wished, and when I first saw her she had for some time ceased to talk. Her altered temper, her fits of passion, the inarticulate sounds which had taken the place of speech, suggested the idea that idiotcy was supervening on the disorder of the brain, which had formed so marked a feature of her illness. In a short time, however, careful observation ascertained that speech had ceased, because the sense of hearing first had failed; that the case was one for the deaf and dumb school, not for an idiot asylum.

The arrest of development, or the positive retrocession of the mental faculties in childhood may be regarded almost invariably as of far less serious import than any manifest perversion of the moral powers. The child who, in spite of intellectual dullness, attaches itself to those about it, and manifests the ordinary childish feelings, is not one concerning whom there is any occasion to despair, or whom judicious training will not do very much to improve. Several circumstances influence the degree of dulling of the mental powers and are to be taken into consideration in forming an estimate of the child's condition, and his capabilities of improvement. In proportion as the original illness was accompanied with convulsions or serious cerebral disturbance, will the subsequent impairment of the intellectual powers be profound, and their recovery slow in taking place. In proportion, too, to the early age at which such illness occurred, will its results be serious; and this not necessarily owing to the gravity of the mischief, but owing to the low state of attainment to which the child had reached when its further advance was interrupted. Thus, for instance, the apparent obscuration of intellect will be greater if it had overtaken the child before it had learnt to speak, than if it had come on later; and though not the less teachable, yet more will remain to be taught in the former case than in the latter; and a child, perfectly capable of improvement, may be passed over as utterly destitute of all capability of intellectual progress, owing to the age at which disease overtook it not being borne in mind. Lastly, it must be remembered that a very large number of children, whose intellectual progress had been arrested at an early age, are allowed to grow up for years without and culture whatever and that as much of their apparent dullness may be due to unintentional neglect as to actual disability.—*Extract from a Clinical Lecture at the London Hospital for Children.*

